# Voluntary salt reduction by food companies in Japan: a practical guide to target-setting and reformulation strategies

**DOI:** 10.1038/s41440-026-02590-z

**Published:** 2026-02-19

**Authors:** Nayu Ikeda, Miwa Yamaguchi, Ikuko Kashino, Katsuyuki Miura, Nobuo Nishi

**Affiliations:** 1 National Institute of Health and Nutrition, National Institutes of Biomedical Innovation, Health and Nutrition, Osaka, Japan; 2 College of Gastronomy Management, Ritsumeikan University, Shiga, Japan; 3 Department of Nutrition, Koshien University, Hyogo, Japan; 4 NCD Epidemiology Research Center, Shiga University of Medical Science, Shiga, Japan; 5 Japanese Society of Hypertension, Tokyo, Japan; 6 Graduate School of Public Health, St Luke’s International University, Tokyo, Japan

**Keywords:** Cardiovascular disease, Food industry, Hypertension, Salt-reduction targets, Voluntary product reformulation

## Abstract

Excessive dietary salt intake remains a major public health concern in Japan and worldwide, contributing to noncommunicable diseases, including hypertension and cardiovascular disease. Although national health promotion strategies in Japan have emphasized behavioral change through nutrition education and awareness campaigns to achieve population-level salt-reduction targets, average intake continues to exceed recommended levels. This suggests that the food environment needs structural modifications through multisectoral collaboration to increase the nutritional quality of consumer foods and the availability of healthier options. Voluntary reformulation by the food industry is a key component of these efforts. To support voluntary reformulation, we developed a practical guide for setting salt-reduction targets and planning feasible reformulation strategies, informed by consultation with registered dietitians working in national and local governments, reviews of guidance documents and voluntary corporate initiatives in other high-income countries, and feedback from Japanese food companies on the draft guide. This guide promotes target setting aligned with the Specific, Measurable, Achievable, Relevant, and Time-bound (SMART) framework for goal-setting and outlines methodological options for product scope, nutrient focus (salt alone, or salt and other nutrients such as fat and sugar), metrics, sodium criteria, and implementation timelines. The guide also addresses organizational structures and collaboration with external stakeholders. Business incentives are highlighted, including opportunities for product innovation, contributions to environmental, social, and governance performance, and the building of consumer trust. By providing a structured and adaptable framework, the guide aims to foster coordinated industry engagement in salt reduction to prevent hypertension and cardiovascular disease.

**Figure:**
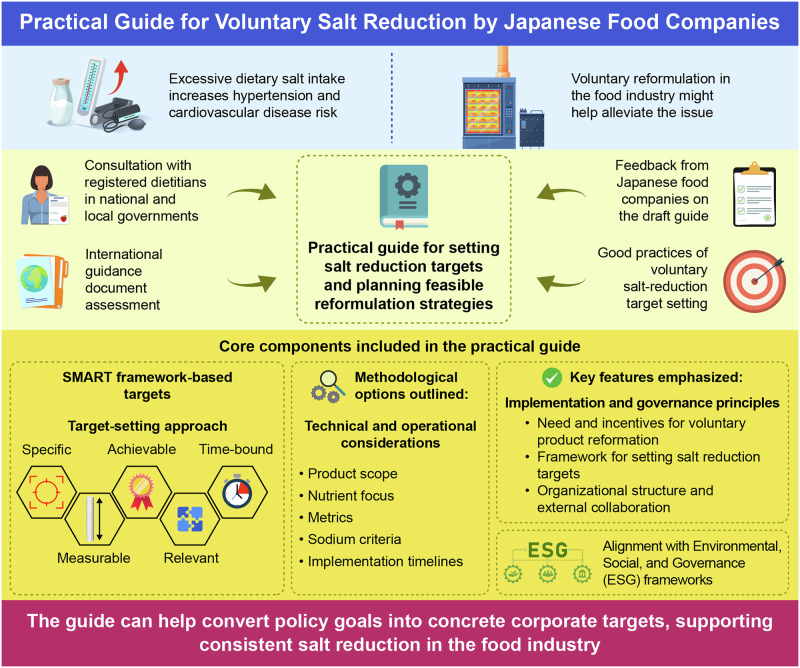
This mini review presents a guide to help Japanese food companies set voluntary salt-reduction targets and plan feasible product reformulation strategies. It integrates international guidance, registered dietitians’ and companies’ feedback, and the SMART framework to help translate policy goals into actionable objectives, promoting consistent salt reduction across the food industry

This mini review presents a guide to help Japanese food companies set voluntary salt-reduction targets and plan feasible product reformulation strategies. It integrates international guidance, registered dietitians’ and companies’ feedback, and the SMART framework to help translate policy goals into actionable objectives, promoting consistent salt reduction across the food industry

## Introduction

Globally, excessive dietary salt intake is a major contributor to noncommunicable diseases, including kidney disease, hypertension, and cardiovascular disease and is a leading dietary risk factor for premature mortality and disability [1]. The World Health Organization (WHO) recommends limiting daily salt intake to <5 g [2]. The WHO originally set a target of a 30% reduction in population-level salt intake between 2013 and 2025 [3], later extending the timeline to 2030 [4]. However, global daily average intake in 2021 was estimated at 10.7 g, and most populations continue to exceed the recommended level [5].

In Japan, salt reduction has long been incorporated into national health promotion strategies. The first (2000–2010) and second (2013–2022) terms of the Health Japan 21 initiative set average daily intake targets of 10 g and 8 g, respectively [6, 7]. These programs endorsed behavioral change through nutrition education and awareness campaigns. Although these efforts coincided with gradual declines in average salt intake from ~14 g in 1995 to 10 g in the mid-2010s, progress has plateaued in recent years [8]. Specifically, the average daily salt intake among Japanese adults in 2023 remained 9.8 g, nearly double the WHO recommendation [9]. This stalled progress may be partly driven by non-discretionary salt intake. A nationwide study showed that in 2013, 40–60% of daily salt intake among adults originated from ready-made foods and dining out, depending on sex and age [10]. In addition, according to the 2023 Japan National Health and Nutrition Survey, a substantial part of average daily salt intake among adults was from processed foods, including soy sauce (1.6 g), soybean paste (1.1 g), processed fish and shellfish (0.6 g), bread (0.4 g), pickled vegetables (0.4 g), ham and sausage (0.3 g), and instant Chinese noodles (0.3 g) [9]. Therefore, education alone is insufficient to achieve population-level salt-reduction goals. Structural modifications to the food environment are essential to ensure healthier food choices.

The Japanese government revised the national average daily salt-intake target to 7 g by the end of fiscal year 2032, as part of the third term of the Health Japan 21 initiative (2024–2035) [11]. Meanwhile, the Strategic Initiative for a Healthy and Sustainable Food Environment was launched in 2022 [12, 13]. This initiative promotes multisectoral collaboration among government, industry, academia, and civil society to enhance the nutritional quality of consumer foods and facilitate healthier choices. Voluntary reformulation is highlighted as a key component of these efforts. Since food companies shape the nutritional profiles of products, they are expected to contribute to improving population-level dietary patterns.

Despite these policy developments and expectations for industry participation, there has been no unified, publicly available framework to guide sector-wide action for voluntary reformulation for salt reduction. While some companies have already reduced the salt content of their products in the market [14], efforts remain fragmented. Small- and medium-sized enterprises may lack the resources and technical capacity to develop effective salt-reduction strategies, particularly when reformulating products for the first time. Therefore, practical guidance is needed to translate public health goals into realistic targets and reformulation plans grounded in common benchmarks and evaluation methods.

We developed a practical guide for Japanese food companies to set salt-reduction targets and plan feasible strategies for voluntary product reformulation in Japanese and English [15–17]. This mini review outlines the methods we used to develop the guide and summarizes its key recommendations and future directions. Although mandatory government-led approaches exist in some countries [15, 18], comparing voluntary and mandatory strategies or presenting specific formulation techniques or recipes falls beyond the scope of the guide and this study.

## Development of the guide

The guide was developed through consultation, evidence gathering, and iterative refinement. We initially consulted registered dietitians working in national and local governments who were involved in policy development for healthier food environments. Through several face-to-face and online meetings, they provided feedback that helped identify key challenges and concerns in promoting voluntary reformulation. Building on their input, we refined the structure and content of the guide to reflect the circumstances of Japanese food companies. Discussions addressed the types of companies to be targeted and practical approaches to setting salt-reduction targets. We elaborated on the use of sales-weighted average sodium content and the application of the Japanese nutrient profiling model. We also examined the importance of labeling reformulated products, specifically whether they are marketed with or without explicit “salt-reduced” claims, and of establishing organizational frameworks to support salt-reduction strategies.

To further inform the framework, we conducted a scoping review of guidance documents from other high-income countries [19], including the United Kingdom [20], Canada [21], the United States [22], and members of the European Union [23]. These documents highlighted common elements relevant to our guide, such as the rationale for developing guidance, current salt-intake levels, national reduction targets, and implementation timelines. We also examined voluntary salt-reduction targets adopted by food companies in other high-income countries to identify common approaches to target setting. The background and methods of this study are described in detail elsewhere [18]. In brief, the work involved a scoping review of published and gray literature and corporate websites to extract information on target-setting frameworks and understand how targets were structured and monitored in different contexts. Additionally, we conducted a survey on incentives and challenges related to voluntary reformulation [18]. Ten food companies and three trade associations in other high-income countries were invited through our professional research networks. Representatives responsible for operational functions related to nutrition and product development from one large multinational company and two national trade associations submitted responses by email. The written questionnaire collected information on (1) target-setting and reformulation activities, (2) incentives from an environmental, social, and governance (ESG) perspective, and (3) challenges and potential solutions. These responses, which provided insights not obtainable through the scoping review alone, were incorporated into the guide.

To ensure the guide’s practical relevance, we sought feedback on draft versions from the registered dietitians consulted earlier. We also conducted a survey with seven Japanese food companies to assess potential demand among their intended users. Invitations to participate were distributed through the registered dietitians, the chairperson of the Salt Reduction and Nutrition Committee of the Japanese Society of Hypertension, and the KARUSHIOH Project secretariat at the National Cerebral and Cardiovascular Center [24]. The questionnaire asked company representatives which business units or personnel would be expected to use the guide and which sections they anticipated would be useful, with an optional open-ended field for additional comments. Feedback from the registered dietitians and survey participants was then incorporated to revise and finalize the guide.

## Contents of the guide

The guide is intended for all Japanese food companies, including those considering salt reduction for the first time and those seeking to reassess or expand existing initiatives. It offers both a conceptual foundation and operational tools that companies can adapt to their circumstances, balancing standardization with flexibility to support diverse product portfolios, organizational structures, and technical capacities.

The content is organized into three chapters. Chapter 1 highlights the importance of voluntary reformulation by the food industry to reduce salt, from both public health and business perspectives. Chapter 2 summarizes key considerations for setting feasible, stepwise salt-reduction targets. Chapter 3 discusses organizational arrangements and collaboration with external stakeholders to support effective salt-reduction initiatives. The appendix presents case studies of voluntary salt-reduction target-setting in Japan and other high-income countries.

The guide promotes target setting aligned with the Specific, Measurable, Achievable, Relevant, and Time-bound (SMART) framework [25]. By providing a systematic structure for defining objectives, tracking progress, and maintaining accountability, it supports companies in developing actionable salt-reduction strategies linked to measurable outcomes.

### Need and incentives for product reformulation

Given Japan’s high average population salt intake, reformulating food products is both necessary and strategically advantageous. Regularly reporting progress toward measurable targets can create a reinforcing cycle, generating opportunities for product innovation, enhanced ESG performance, stronger investment appeal, increased consumer trust, and growth in sales of low-salt products.

### Framework for salt-reduction target-setting

The guide outlines relevant national and global intake goals as the foundation for setting corporate salt-reduction targets. These include the Dietary Reference Intakes for Japanese (2025 edition; <7.5 g and <6.5 g per day for men and women, respectively) [26], the Japanese Society of Hypertension Guidelines (JSH2025; <6 g per day for patients with hypertension) [27], and the WHO recommendation (<5 g per day) [2]. The guide explains how companies can reference these goals when setting reformulation targets according to their business circumstances. Several key dimensions are identified, such as target product scope, nutrient focus, target metrics, and sodium criteria. Various reformulation approaches are outlined across these dimensions (Table 1) [18, 28]. These approaches can be combined to balance salt-intake goals with business feasibility.

**Table 1:** Methods for setting salt-reduction targets

Dimensions	Methods
Target product scope	• Food product • Food category • Entire product portfolio
Nutrient focus	• Salt alone • Salt and other nutrients (e.g., fat and sugar)
Target metrics	• Maximum limit • Simple average • Sales-weighted average • Relative reduction
Sodium criteria^1^	• Nutrient profiling model • Sodium-specific criteria

^1^ Sodium criteria could be developed externally by government authorities or internally by companies

The guide provides further detail on several methodological options for setting targets. A sales-weighted average (i.e., calculating the average salt content of products weighted by sales volume) is recommended as a target metric because it prioritizes high-selling items. Examples of sales-weighted average calculations are provided for high-salt Japanese food categories such as miso, soy sauce, pickles, and processed seafood. With respect to externally defined sodium criteria, the guide refers to the Japanese nutrient profiling model, which is available for both processed foods and dishes [29, 30], and WHO global sodium benchmarks [31]. In addition, it describes how to calculate relative salt reduction (i.e., total amount of salt reduced through sales of the reformulated vs. original product), drawing on examples from low-salt foods certified by the Japanese Society of Hypertension [14]. Finally, because implementing evaluations in practice can be time-consuming and costly, the guide suggests using simulation modeling as an alternative to assess potential impacts of reformulation on population-level salt intake.

### Implementation timelines

Implementation timelines are integral to effective salt-reduction strategies. International data shows that gradual reformulation is technically feasible and more acceptable to consumers, as taste preferences adapt progressively [18]. The guide, therefore, stresses the importance of setting company-level timelines that allow for gradual reformulation and align with broader public health milestones, such as the 2030 target specified in the Sustainable Development Goals [32] or national dietary goals, to ensure coherence between corporate initiatives and policy agendas. To maintain flexibility and encourage long-term engagement, the guide recommends establishing time-bound targets that can be updated in light of new scientific evidence, market dynamics, or technological advances. The selection of achievement years should reflect the scale of the product category and the extent of salt reduction required.

To balance feasibility with consumer acceptance, the guide advises the use of interim evaluation points and staged reformulation. For example, a company could commit to reducing the salt content of a flagship product line within three years, while establishing checkpoints to monitor progress, evaluate consumer responses, and make adjustments.

### Organizational structure and external collaboration

The guide outlines two organizational models for effective salt-reduction initiatives. In the first model, the product planning and development division leads reformulation efforts and target-setting, with senior management approving the proposed strategy before implementation. The second model is suited to companies that lack sufficient internal capacity. It recommends participation in trade associations and the sharing of common goals to obtain external support, peer learning, and a foundation for gradual progress.

The guide also emphasizes that external collaboration can enhance feasibility and credibility. Partnerships are recommended with the Ministry of Health, Labour and Welfare’s Strategic Initiative for a Healthy and Sustainable Food Environment [12], consultation with the Japanese Society of Hypertension’s Salt Reduction and Nutrition Committee for technical advice and product certification [33], and cooperation with the National Cerebral and Cardiovascular Center’s KARUSHIOH Project, which promotes flavor enhancement as a key salt-reduction strategy [24].

### Technical approaches to salt reduction

The guide outlines several technical salt-reduction strategies that maintain product quality, including partial replacement of sodium chloride with potassium chloride and the use of flavor-enhancing vegetables, herbs, and spices [34–39]. The guide includes a case box with reformulation examples as a practical illustration, designed to reduce salt without compromising palatability, product safety, or shelf life.

### Case studies

The case studies in the guide’s appendix illustrate practical approaches to setting salt-reduction targets. Companies were selected based on the availability of publicly accessible information on product scope, sodium standards, and implementation timelines in recent integrated reports or on official websites.

### Industry feedback

Responses to the company survey on the draft guide indicated that the guide would be most relevant to product planning and public relations departments. Some respondents valued its usefulness in setting reformulation goals, developing medium- to long-term product strategies, and communicating with consumers and stakeholders. However, others pointed out the limited scope for salt reduction in certain products owing to manufacturing and preservation requirements, challenges in interpreting the relevance of results from studies conducted in other countries to the Japanese context, and the importance of consumer education as a complementary measure. The draft guide was revised to reflect this feedback.

## Discussion

To the best of our knowledge, this guide is the first to compile scientific evidence and policy information for food companies considering setting salt-reduction targets in Japan. Aligned with the national initiative for healthier food environments, the guide promotes target setting as per the SMART framework, highlights incentives, including improved ESG performance for investment, and integrates the sales-weighted average sodium content and nutrient profiling models adapted to Japanese dietary patterns.

This guide represents a significant step in broader efforts to transform food systems for sustainable development. It offers a coherent framework for structured target setting, grounded in scientific evidence, policy directions, and industry input. Companies of varying sizes and capacities can adapt the framework to their circumstances. The guide was developed to encourage industry engagement and is intended for companies pursuing, planning, or revising salt-reduction strategies.

The implications extend beyond individual companies. By presenting common reference points, methodological options, and organizational approaches, the guide establishes a foundation for greater consistency across the food sector. This alignment could strengthen links between corporate initiatives and national strategies, while also creating opportunities to integrate reformulation efforts into corporate agendas, including ESG performance and consumer trust. From a public health perspective, the guide signals a shift toward more systemic private-sector engagement in addressing dietary risk factors to reduce the burden of noncommunicable diseases.

Challenges remain in ensuring the guide’s practical applicability. Although the framework is broadly applicable across company sizes and product portfolios, adoption may be less likely among producers of food items with inherently high salt content for safety or preservation purposes, or among those for whom salt reduction is a low priority. Some companies, particularly nationwide operators, may require more detailed, practice-oriented guidance.

Robust and consistent metrics are essential for tracking progress in salt-reduction efforts. Given the absence of comprehensive data on the salt content of domestic products, particularly sales-weighted averages, and the ongoing refinement of the Japanese nutrient profiling model, the guide does not prescribe specific numerical targets. Instead, it emphasizes a stepwise, adaptable approach to goal-setting, tailored to the unique circumstances of each company. Therefore, the guide should be viewed as a conceptual framework for salt-reduction target setting, rather than as a set of prescriptive targets.

The industry survey suggested that the guide is particularly useful for product planning and public relations departments, highlighting the importance of building shared recognition within companies. However, the same feedback underscored concerns about the economic burden of developing and manufacturing reduced-salt foods, such as maintaining taste and ensuring food safety. These costs may be translated into higher prices, potentially discouraging consumer purchase of reduced-salt products. Dialog between governments and the food industry is therefore essential to ensure that incentives for reformulation outweigh these costs. Future revisions of the guide should incorporate such stakeholder engagement, tailor guidance according to company size and stage of reformulation, and include real-world case studies as more companies implement salt-reduction initiatives.

Information on the guide’s publication has been disseminated through related channels, including an institutional press release [40], to support awareness and uptake. Future research could evaluate the guide’s effectiveness, for example, by surveying companies on adoption and potential impacts on the salt content of foods.

In summary, the guide should be regarded as a living framework that evolves with new data, methodological advances (e.g., nutrient profiling models), and practical implementation experiences. Its future contribution will depend on sustained dialog among industry, government, academia, and civil society, supported by transparent evaluation and reporting of outcomes. As it is refined and more widely adopted, the guide may accelerate Japan’s progress toward national salt-reduction goals and contribute to lowering the burden of hypertension and cardiovascular disease.
